# Profiling Antibiotic Susceptibility among Distinct *Enterococcus faecalis* Isolates from Dental Root Canals

**DOI:** 10.3390/antibiotics13010018

**Published:** 2023-12-24

**Authors:** Daniel Manoil, Ender Efe Cerit, Hong Fang, Stéphane Durual, Malin Brundin, Georgios N. Belibasakis

**Affiliations:** 1 Division of Cariology and Endodontics, University Clinics of Dental Medicine, Faculty of Medicine, University of Geneva, 1211 Geneva, Switzerland; 2 Division of Oral Health and Periodontology, Department of Dental Medicine, Karolinska Institute, Campus Huddinge, 141 52 Stockholm, Sweden; enderefe@hotmail.com; 3 Department of Laboratory Medicine, Karolinska University Hospital Huddinge, Karolinska Institute, Campus Huddinge, 141 52 Stockholm, Sweden; hong.fang@regionstockholm.se; 4 Biomaterials Laboratory, Division of Fixed Prosthodontics and Biomaterials, University Clinics of Dental Medicine, Faculty of Medicine, University of Geneva, 1211 Geneva, Switzerland; stephane.durual@unige.ch; 5 Division of Endodontics, Department of Odontology, Umeå University, 901 87 Umeå, Sweden; malin.brundin@umu.se

**Keywords:** *Enterococcus faecalis*, antimicrobial susceptibility testing, antibiotic resistance, endodontic infections, vancomycin-resistant enterococci, tigecycline-resistant VRE

## Abstract

*Enterococcus faecalis*, a leading multi-resistant nosocomial pathogen, is also the most frequently retrieved species from persistently infected dental root canals, suggesting that the oral cavity is a possible reservoir for resistant strains. However, antimicrobial susceptibility testing (AST) for oral enterococci remains scarce. Here, we examined the AST profiles of 37 *E. faecalis* strains, including thirty-four endodontic isolates, two vanA-type vancomycin-resistant isolates, and the reference strain ATCC-29212. Using Etest gradient strips and established EUCAST standards, we determined minimum inhibitory concentrations (MICs) for amoxicillin, vancomycin, clindamycin, tigecycline, linezolid, and daptomycin. Results revealed that most endodontic isolates were susceptible to amoxicillin and vancomycin, with varying levels of intrinsic resistance to clindamycin. Isolates exceeding the clindamycin MIC of the ATCC-29212 strain were further tested against last-resort antibiotics, with 7/27 exhibiting MICs matching the susceptibility breakpoint for tigecycline, and 1/27 reaching that of linezolid. Both vanA isolates confirmed vancomycin resistance and demonstrated resistance to tigecycline. In conclusion, while most endodontic isolates remained susceptible to first-line antibiotics, several displayed marked intrinsic clindamycin resistance, and MICs matched tigecycline’s breakpoint. The discovery of tigecycline resistance in vanA isolates highlights the propensity of clinical clone clusters to acquire multidrug resistance. Our results emphasize the importance of implementing AST strategies in dental practices for continued resistance surveillance.

## 1. Introduction

*Enterococcus faecalis* is a Gram-positive facultatively anaerobic coccus that naturally inhabits the gastrointestinal tracts of humans, dogs, cats, poultry, and a variety of insects, and can thrive in environments tainted by human and animal fecal matter [1]. The species is sturdy, metabolically versatile, and has evolved to sustain harsh conditions, including high salt concentrations, varying temperatures (from 10 to >45 °C), and high pH or oxidative stresses [2,3,4,5]. Moreover, *E. faecalis* is inherently resistant to several antibiotics such as clindamycin, metronidazole, or aminoglycosides [6]. Such resilience endows *E. faecalis* with a remarkable opportunistic pathogenic potential; the genus represents the third most frequently identified pathogen in healthcare-associated infections and is the second leading cause of septicemia in intensive care units [7,8]. 

Apart from its involvement in nosocomial infections, *E. faecalis* is also found in the oral microbiome. However, because it is often reported in low abundances in community profiling studies [9,10], its ecological role and potential pathogenicity remain incompletely understood. Various reports suggest the species to be allochthonous to the oral microbiome, possibly foodborne via dairy consumption [11,12,13]. Conversely, other reports have consistently associated *E. faecalis* with oral diseases, especially endodontic infections, suggesting a causal contribution of the species [14]. Indeed, whereas endodontic infections are polymicrobial in nature, *E. faecalis* is one of the most frequently detected species in dental root canals with post-treatment apical periodontitis [15,16,17]. Notably, several microbial profiling studies have reported remarkably high relative abundances of *E. faecalis* in these infected canals, possibly constituting anywhere from 14% to 99% of the infecting communities [18,19,20].

The occurrence of *E. faecalis* within infected roots appears significantly associated with the presence of periapical lesions, thereby increasing the odds of local and systemic infectious exacerbations of these teeth [21,22]. Whereas such infections are likely to require local and/or systemic antimicrobial therapy, only limited data on antimicrobial susceptibility testing (AST) are available for enterococcal endodontic isolates, even when considering commonly employed antibiotics in dental medicine [23,24,25,26]. The paucity of AST data is even more pronounced for more recent antibiotics, such as tigecycline, linezolid, or daptomycin. Gathering such data is of increasing relevance due to the rise in *E. faecalis* isolates exhibiting resistance against these last-resort antibiotics [27,28]. Box 1 presents several key concepts that are integral to understanding antimicrobial susceptibility testing outcomes. Additionally, the prominent presence of *E. faecalis* within root canals also prompts the question of the oral cavity potentially serving as a reservoir for resistant *E. faecalis* isolates [22,29]. Given *E. faecalis*’ unique ability to acquire and disseminate determinants of antibiotic resistance [30], the presence of resistant isolates in polymicrobial ecosystems, such as the oral cavity, could contribute to enriching the pool of latent antibiotic resistance genes present within the oral microbiome [31]. 

Therefore, in this report, we aimed to screen the antibiotic susceptibility profiles of various endodontic *E. faecalis* isolates. More specifically, we employed Etest strips to determine the minimum inhibitory concentration (MIC) values of six antibiotics. These included commonly used antibiotics in dental medicine, such as amoxicillin and clindamycin, as well as antibiotics whose resistance acquisition is linked with increased morbidity and mortality, namely vancomycin, tigecycline, linezolid, and daptomycin.

Box 1 **Key concepts to understand antimicrobial susceptibility testing (AST).**

The **minimum inhibitory concentration (MIC)** represents the lowest concentration of an antibiotic necessary to inhibit the growth of a **specific** microorganism [32]. By determining a MIC value, microbiologists assess the potency of an antibiotic against a particular bacterial taxon, which is paramount to devising effective antimicrobial therapies [33].

The **epidemiological cut-off (ECOFF)** is an essential parameter used in AST. A MIC distribution is initially acquired from several isolates of the same bacterial taxon exposed to one particular antibiotic. A typical distribution plots increasing concentrations of that antibiotic on the x-axis against the number of tested isolates on the y-axis. The ECOFF then designates the upper limit of this MIC distribution. ECOFF determination assumes that no acquired or mutational resistance mechanisms are present. Essentially, the ECOFF value determines the threshold between wild type susceptible strains and those with acquired resistance [34]. ECOFFs are statistically determined from collated data derived from thousands of AST distributions [35]. The European Committee on Antimicrobial Susceptibility Testing (EUCAST) mostly relies on statistical programs—such as ECOFFinder—to determine an ECOFF value [36]. The use of ECOFFs is crucial for monitoring the development of new resistances and setting clinical breakpoints [37].

A **breakpoint** is a set MIC value that categorizes bacterial isolates into three categories: “S—susceptible”, “I—Susceptible, yet increased exposure required”, and “R—resistant” [38]. If the MIC for a bacterial isolate is lower than the breakpoint, the taxon is considered susceptible to the antibiotic, which is, therefore, likely to be used effectively. If the MIC is higher, the bacterium is deemed resistant. Breakpoints are determined by expert committees, such as the EUCAST, that comprehensively integrate multiple datasets, including pharmacokinetics and pharmacodynamics, clinical efficacy, toxicity and dosages, MIC distributions, and the prevalence of resistance mechanisms [39]. Breakpoints are regularly reviewed and updated to provide critical guidance for therapeutic decisions.

## 2. Results

### 2.1. AST Outcomes for Amoxicillin, Vancomycin, and Clindamycin

**Figure 1:**
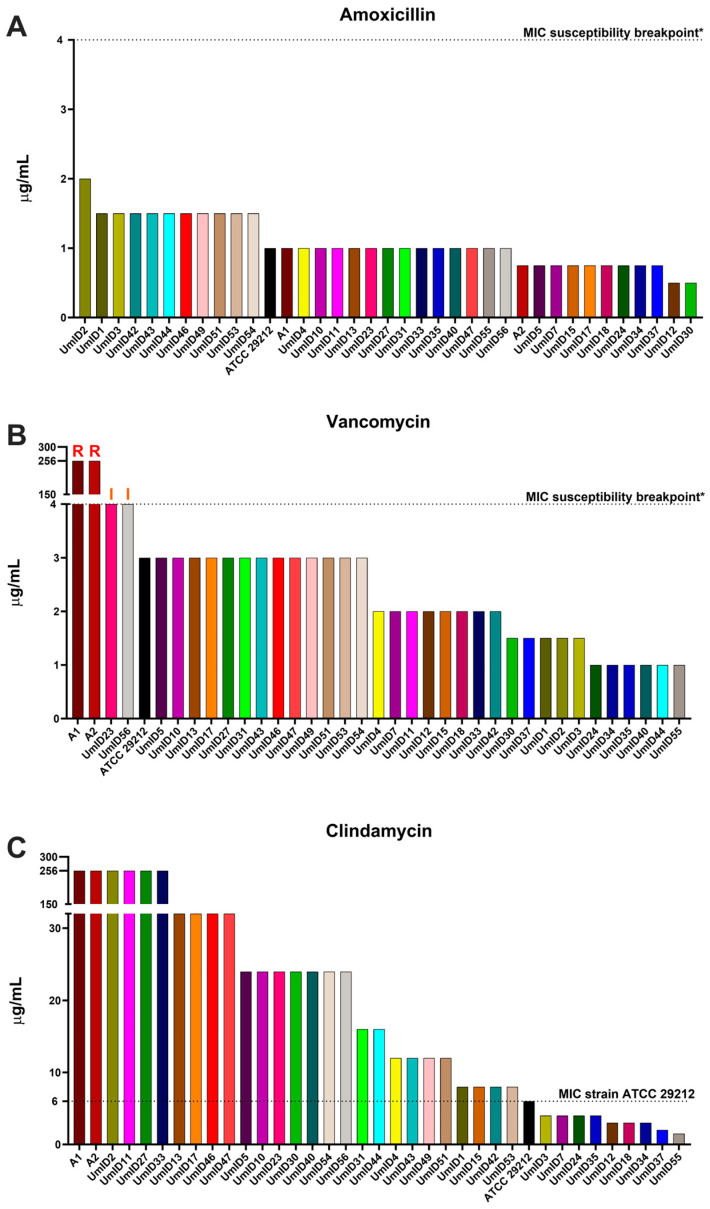
**AST values of thirty-seven *E. faecalis* strains for three frequently prescribed antibiotics.** Histograms show the MIC values expressed in μg/mL (y-axis) in each strain of *E. faecalis* tested (x-axis). Amoxicillin is shown in (**A**), vancomycin is shown in (**B**), and clindamycin is shown in (**C**). MICs for vancomycin and clindamycin are displayed on a split y-axis that covers values from 0 to 4 μg/mL on its lower segment and from 150 to 256 μg/mL on its upper segment. For each antibiotic, the dotted line represents the MIC susceptibility breakpoint (S≤; R>). No MIC susceptibility breakpoint is provided for clindamycin because *E. faecalis* is considered intrinsically resistant to lincosamides. *: MIC susceptibility breakpoint values were extracted from the EUCAST table v_13.1 (https://www.eucast.org/clinical_breakpoints) (URL accessed on 30 November 2023). Above bars: R marks resistant MICs and I indicates MICs that reach the clinical breakpoint that could be considered “I—Susceptible, Increased exposure”.

Figure 1 presents MIC values for three antibiotics—amoxicillin, vancomycin, and clindamycin—tested on thirty-seven *E. faecalis* strains. In the case of amoxicillin (Figure 1A and Supplementary Table S1), the reference strain ATCC 29212 and the two vanA isolates (labeled A1 and A2) exhibited susceptible MIC values ranging between 0.75 and 1 µg/mL, i.e., below the 4 μg/mL breakpoint. Similarly, all 34 endodontic isolates demonstrated MIC values falling within the susceptibility range. The observed MIC values spanned a modest range, with a minimum of 0.5 µg/mL and a maximum of 2 µg/mL.

For vancomycin (Figure 1B), the ATCC 29212 strain displayed a MIC value predictably within the ECOFF range of the species (3 μg/mL), while the two resistant vanA isolates largely exceeded the susceptibility breakpoint of 4 μg/mL, each displaying a MIC of 256 μg/mL. Similar to the reference ATCC 29212, the majority of endodontic isolates exhibited values within the ECOFF range, but UmID23 and UmID56 reached the susceptibility breakpoint and were categorized as “I—Susceptible, Increased exposure”.

Because clindamycin ranks among the most frequently prescribed antibiotics for odontogenic infections, we deemed valuable to assess AST outcomes in our *E. faecalis* collection, despite its intrinsic resistance. The reference ATCC 29212 strain displayed a MIC of 6 μg/mL (Figure 1C). Nine endodontic isolates exhibited MIC values below the ATCC 29212 reference. Among these, two isolates—UmID37 and UmID55—exhibited MICs of 2 and 1.5 μg/mL, respectively, indicative of low-level intrinsic resistance. Conversely, the remaining 27 isolates—which included the two *vanA* carriers—exhibited high-level intrinsic resistance, with MICs ranging from 8 to a substantial 256 μg/mL. Because clindamycin resistance in *E. faecalis* may encompass various distinct mechanisms, some of which could potentially bestow cross-resistance to critical last-resort antibiotics, such as linezolid [40,41,42], we conducted additional AST assays on these 27 isolates that exhibited high-level intrinsic resistance. These 27 isolates were subjected to AST assessments using tigecycline, linezolid, and daptomycin, all of which are considered last-resort antibiotics for treating enterococcal infections [28].

### 2.2. AST Outcomes in E. faecalis Isolates with High Clindamycin Intrinsic Resistance

**Figure 2:**
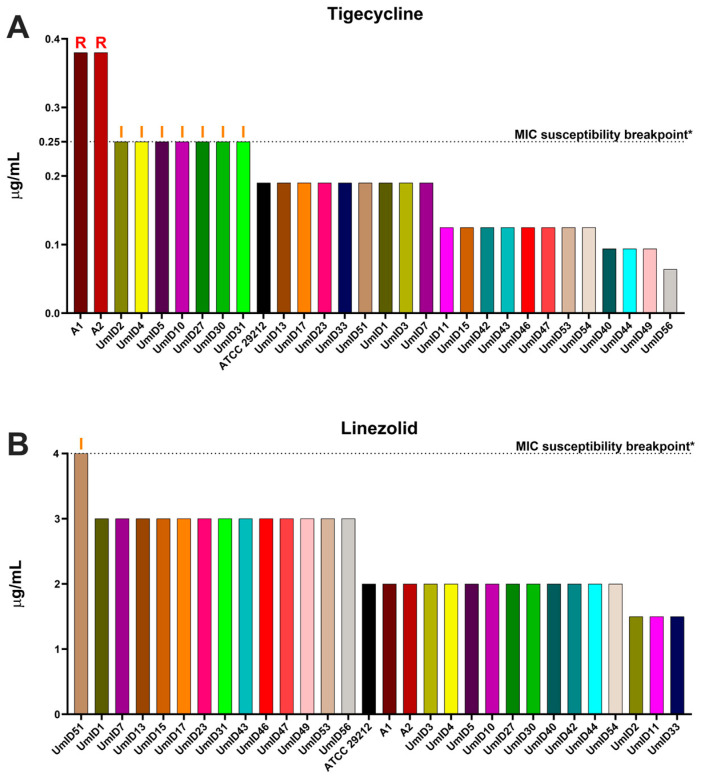

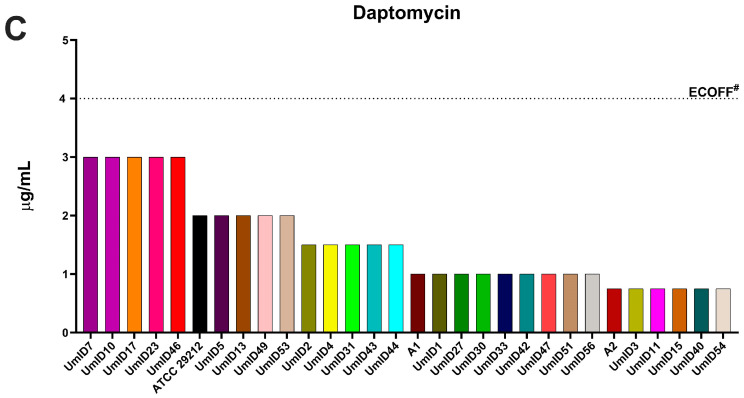
**Tigecycline, linezolid, and daptomycin AST values for *E. faecalis* strains with high intrinsic resistance to clindamycin.** Histograms show the MIC values expressed in μg/mL (y-axis) of each strain of *E. faecalis* tested (x-axis). Tigecycline is shown (**A**), linezolid is shown in (**B**), and daptomycin is shown in (**C**). For each antibiotic, the dotted line represents the MIC susceptibility breakpoint (S≤; R>). The *E. faecalis*’ ECOFF is provided for daptomycin instead of a MIC breakpoint. This is because the EUCAST Steering Committee currently considers that there is insufficient evidence to determine a breakpoint for daptomycin [43]. *: MIC susceptibility breakpoint values were extracted from the EUCAST table v_13.1 (https://www.eucast.org/clinical_breakpoints) (URL accessed on 30 November 2023). ^#^: The daptomycin ECOFF value was extracted from (https://www.eucast.org/mic_and_zone_distributions_and_ecoffs/new_and_revised_ecoffs) (URL accessed on 30 November 2023). Above bars: R marks resistant MICs and I indicates MICs that reach the clinical breakpoint that could be considered “I—Susceptible, Increased exposure”.

Figure 2 displays the AST outcomes for tigecycline, linezolid, and daptomycin. In the case of tigecycline, whose breakpoint for *E. faecalis* is 0.25 μg/mL, the reference strain ATCC 29212 exhibited a susceptible MIC value at 0.19 μg/mL. Similarly, most endodontic isolates (20/27) demonstrated susceptibility to tigecycline, with MIC values substantially below the susceptibility breakpoint (0.25 μg/mL) (Figure 2A). In contrast, seven endodontic isolates showed MIC values borderline with the susceptibility breakpoint, and were, therefore, categorized as “I—Susceptible, Increased exposure”. Most notably, the two *vanA* carrier isolates displayed MIC values of 0.38 μg/mL, i.e., above the MIC breakpoint, thereby demonstrating tigecycline resistance.

In the case of linezolid, the reference strain ATCC 29212 exhibited a MIC of 2 μg/mL, i.e., below the 4 μg/mL breakpoint. Nearly all endodontic isolates displayed similarly susceptible MIC values, showing closely comparable MICs with minor variation between different isolates; the lowest MIC observed was 1.5 μg/mL, while the highest reached 3 μg/mL (Figure 2B). Only one endodontic isolate—UmID51—demonstrated a MIC value that matched the susceptibility breakpoint (4 μg/mL), leading to its classification as “I—Susceptible, Increased exposure”.

For daptomycin, the reference strain ATCC 29212, along with the two *vanA* carriers and all endodontic isolates, demonstrated MIC values that fell within the typical epidemiological range for the species (Figure 2C) and below the 4 μg/mL breakpoint. We note that the EUCAST Steering Committee has acknowledged the absence of sufficient evidence to clearly define a susceptibility breakpoint for this antibiotic. Therefore, we displayed the ECOFF value as a provisional reference point for our observations.

## 3. Discussion

**Figure 3:**
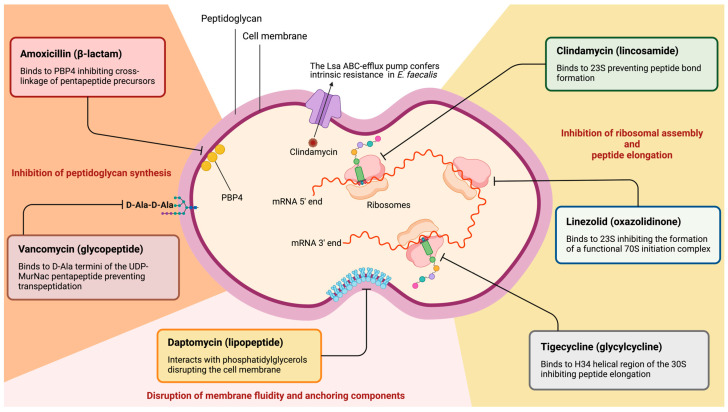
**Mode of action of the antibiotics assessed against *E. faecalis*.** The scheme illustrates the distinct antibacterial mechanisms of the antibiotics assessed in this study. Antibiotics are didactically divided into those that inhibit the synthesis of the peptidoglycan layers (in orange on the left), those that affect the fluidity and function of the cell membrane (in pale pink on the bottom), and those that inhibit ribosomal assembly and elongation of the peptide chain (in yellow on the right). **Amoxicillin** exerts its antimicrobial effect by binding to and inhibiting PBP4, whose role is to polymerize (transglycosylation) and cross-link (transpeptidation) glycan strands between them to produce a functional peptidoglycan wall [44]. **Vancomycin** elicits its effect by binding to D-ala-D-ala terminal moieties of the UDP-MurNac pentapeptide, thereby inhibiting its transpeptidation within the peptidoglycan polymer [27]. **Daptomycin** is a cyclic lipopeptide that partitions into the cytoplasmic membrane at specific sites enriched with phosphatidyl and diphosphatidyl glycerols, resulting in alterations to the membrane fluidity and disruptions of anchorage sites for membrane-bound enzymes and cytoplasmic leakage [27,45,46]. **Tigecycline** binds reversibly to the H34 helical region of the 30S ribosomal subunit and inhibits the incorporation of amino acid residues into the peptide chain [47]. **Linezolid** binds to the 23S ribosomal RNA of the 50S subunit and prevents the formation of a functional 70S initiation complex, thereby inhibiting mRNA translation [48]. **Clindamycin** binds to the 23S portion of the 50S ribosomal subunit and prevents the elongation of the polypeptide chain, thereby hindering protein synthesis [41]. **D-ala**: D-alanine; **UDP-MurNac**: uridine diphosphate N-acetylmuramic acid; **PBP4**: penicillin-binding protein 4. This figure was designed using BioRender.com’s web interface.

This study assessed the AST outcomes of a collection of endodontic *E. faecalis* isolates to three first-line antibiotics—amoxicillin, vancomycin, and clindamycin—and extended this assessment in highly resistant clindamycin isolates to three last-resort antibiotics—tigecycline, linezolid, and daptomycin. Our results demonstrate that most *E. faecalis* endodontic isolates are susceptible to amoxicillin and vancomycin but demonstrated varied levels of resistance to clindamycin. Among those isolates that showed high clindamycin resistance, several displayed borderline MIC values to the susceptibility breakpoints for tigecycline and linezolid. Notably, the two *vanA* carrier isolates phenotypically confirmed high-level vancomycin resistance and demonstrated tigecycline resistance. The mode of action of all antibiotics investigated herein against *E. faecalis* is didactically depicted in Figure 3.

The importance of AST screening in endodontic isolates of *E. faecalis* is manifold. As a member of the microbiota found in recalcitrant endodontic infections, the species may be responsible for infectious exacerbations that invade the neighboring tissues [49]. Whereas the endodontic debridement of such infections is fundamental, successful management frequently incorporates systemic antibiotherapy [50]. Indeed, exacerbated endodontic infections constitute one main cause of dental emergencies [51], pose a risk of expansion to adjacent facial spaces [50], and account for nearly 7000 hospitalizations annually in the US alone [52]. Beyond its role in endodontic infections, monitoring the AST profiles of oral *E. faecalis* may help understand the transmission of resistant isolates. Evidence shows that the oral cavity is likely the primary entry point for acquiring resistant *E. faecalis* strains, which may subsequently establish themselves in the gut microbiota [30,53]. This dynamic holds significant implications, as these “gut” isolates were identified as the main culprits for bacteremias and endocarditis via translocation to the bloodstream or urinary tracts and catheter infections through fecal contamination [30]. Altogether, these observations exemplify how the oral cavity may serve as a dissemination route for resistant taxa and highlight the importance of AST monitoring of oral species [29,31].

Amoxicillin is frequently the first choice for treating bacterial infections in the oral cavity due to its broad spectrum activity against typical oral pathogens and its favorable pharmacokinetics [54,55]. The most frequent resistance mechanism in *E. faecalis* involves mutations that simultaneously alter PBP4′s affinity for amoxicillin and overexpress the modified enzyme [56,57]. In this study, our AST outcomes showed that all tested strains were susceptible to amoxicillin and displayed minimal variations between them. Our observations align with previous studies that report a majority of *E. faecalis* isolates worldwide to be susceptible to β-lactams, such as ampicillin and amoxicillin [23,24,25,26,58,59]. One plausible explanation for such broad susceptibility may be that the induction of resistance imposes a fitness cost deemed “too-high-to-bear”. This is supported by evidence that shows mutated PBP4 variants to be less stable than their native counterparts [60].

Our vancomycin AST outcomes revealed that most strains, including the reference ATCC 29212, fell within the ECOFF range of the species. We confirmed, nonetheless, phenotypical vancomycin resistance in the two vanA isolates. This observation is consistent with previous findings by our group, which highlighted both the presence and functional transcription of vanA operons in these strains [61]. VanA-mediated vancomycin resistance relies on the substitution of D-ala terminal moieties in the UDP-MurNac pentapeptide with D-lactate, which exhibit up to 1000-fold less affinity to vancomycin. Notably, within the endodontic collection examined in this study, two endodontic isolates—UmID23 and UmID56—displayed MIC values that matched the EUCAST susceptibility breakpoint. This suggests that effective inactivation of these strains may necessitate elevated clinical concentrations of vancomycin, although strict resistance could not be identified. 

In this study, our *E. faecalis* collection was also subjected to AST for clindamycin. Despite the intrinsic resistance of *E. faecalis* to lincosamides, we deemed these AST assessments to be valuable since clindamycin ranks as the second most prescribed antibiotic in dental medicine, often preferred in cases of hypersensitivity to penicillins [55]. This means that clindamycin is often employed to treat polymicrobial oral infections, possibly leading to the suboptimal exposure of *E. faecalis* cells, which would then be prone to overtake the infected site. In the present study, the reference strain ATCC 29212 yielded a MIC of 6 μg/mL, which may be didactically viewed as a “benchmark intrinsic resistance”. Nine endodontic isolates of our collection exhibited MIC values below that “benchmark”, among which UmID37 and UmID55 displayed remarkably low-level intrinsic resistance, with MICs of 2 and 1.5 μg/mL, respectively, approaching those of taxonomically related, clindamycin-susceptible taxa. For context, the EUCAST reports MIC susceptibility breakpoints of 0.25 μg/mL for *Staphylococcus aureus* and 0.5 μg/mL for streptococci A, B, C, and G. In contrast, 25 of our isolates displayed MICs above the ATCC 29212 “benchmark”, ranging from 8 to over 256 μg/mL. Such a broad spectrum of AST outcomes is consistent with previously reported phenotypical variations in clindamycin resistance [24,25,62]. Indeed, whereas clindamycin resistance in *E. faecalis* is predominantly attributed to the *lsa* gene, which encodes an ABC efflux pump on the core genome, evidence has shown variability in pump activity, and in rare cases, even a lack of functionality [24]. Worth noting is that although clindamycin resistance in *E. faecalis* could also result from the transferable *cfr* gene (chloramphenicol–florfenicol resistance), which methylates the 23S rRNA, this would confer cross-resistance to linezolid [40,42]. As all isolates in our collection were linezolid-susceptible, our findings suggest an absence of a functional *cfr* gene, although this was not genetically validated in the current study.

Our AST assessments also included tigecycline, a last-resort glycylcycline that displays a 20-fold higher affinity for the 30S ribosomal subunit than tetracycline [40,63]. Initial surveillance post-tigecycline introduction indicated an absence of resistance in *E. faecalis* [64]. In the current study, most of our endodontic isolates demonstrated susceptibility to tigecycline. However, our AST outcomes also revealed that the two *vanA* carrier isolates displayed resistant MICs of 0.38 μg/mL, surpassing the established 0.25 μg/mL EUCAST breakpoint. Although tigecycline resistance is infrequent, with a prevalence of 0.3–0.4% of *E. faecalis* isolates, our findings are noteworthy as they add up to a gradually rising trend of resistance [28,65,66]. Alarmingly, tigecycline resistance seems to preferentially emerge in *vanA* carrier isolates, which inherently resist teicoplanin [67]. Our findings align with another report that identified an outbreak of tigecycline resistance in a hospital-derived clonal cluster of vanA isolates [68]. Taken together, these results add to the growing body of evidence suggesting an association between the vanA operon and resistance to multiple antibiotics, underscoring the challenge of managing infections with multi-resistant strains. It is relevant to mention that the exact mechanism driving tigecycline resistance in *E. faecalis* remains only partially resolved. Current understanding points toward the up-regulation of the TetL efflux pump and the TetM ribosomal protection protein. Moreover, mutations in the *rpsJ* gene, which encodes a ribosomal structural protein, have been observed post-tigecycline exposure [68,69].

It is worth mentioning that whereas our endodontic *E. faecalis* collection is descriptive of the circulating isolates prevailing at the time of their collection (2000–2014), there is evidence showing the evolution of AST profiles and the acquisition of new resistance genes over time [70]. Although this may be perceived as a limitation of the study, it is worth emphasizing that this 15-year timeframe is likely stretched enough to capture a representative landscape of the resistances prevalent in *E. faecalis* endodontic isolates at that time. This is supported by comparisons with analogous studies of the same period, which reveal notable similarities between our AST profiles and those of other oral isolates from Brazil, Finland, Lithuania, and the Netherlands. Specifically, when comparing identical antibiotics across studies, one observes consistent susceptibility to amoxicillin and a similar distribution of the *lsa* gene, conferring clindamycin resistance [23,24,25,26,62]. This knowledge enables the future targeted exploration of clinically relevant genes to better understand their dissemination pathways and decipher the molecular mechanisms driving resistance, particularly when these remain elusive, such as for tigecycline. Finally, depositing AST profiles spanning 2000-14 remains crucial to allow comparisons with more contemporary isolates, offering valuable insights into the dynamics of resistance dissemination among clinical clusters and across various geographical localizations [71,72].

Culture-based AST, as employed in this study, remains a mainstay in clinical microbiology due to its proven efficacy for phenotypic characterization, established standards, and relative ease of use. Yet, it carries inherent limitations that are exposed in several clinical situations, some of which are especially relevant to our study and the oral ecosystem. Notably, phenotypic approaches do not inform on potentially carried resistance genes that are not expressed. Typically, certain *vanA* carrier enterococci were shown to exhibit phenotypic susceptibility to vancomycin while retaining the ability to revert to a vancomycin-resistance phenotype, designated as vancomycin-variable enterococci—VVE [73]. Also, outcomes of phenotypic AST are highly dependent on specific growth requirements and are, therefore, likely to miss not readily culturable taxa in a sample, such as commonly found in the oral microbiome or polymicrobial infections. Finally, because culture-based methods require the growth of bacteria, they may sometimes extend beyond 72 h. Such a time-consuming approach may cause serious setbacks when rapid guidance for antibiotherapy is required. 

To conclude, our findings demonstrate that while the majority of our endodontic collection remained susceptible to first-line antibiotics, such as amoxicillin and vancomycin, several isolates exhibited marked intrinsic resistance to clindamycin and MIC values that matched the breakpoints of last-resort antibiotics, such as tigecycline. The emerging tigecycline resistance in vanA isolates emphasizes the association between the presence of vanA operons and the acquisition of other resistance genes. Collectively, these results underline the importance of continued resistance surveillance. They also highlight the value of implementing rapid and accurate AST strategies in dental clinical settings. Doing so may not only enhance patient care but also help monitor the transmission of antibiotic resistance genes in hospital settings.

## 4. Materials and Methods

### 4.1. Bacterial Strains and Isolates

In this study, a total of 37 strains of *E. faecalis* were included. The strain ATCC 29212 served as the reference strain, and two vanA-type vancomycin-resistant clinical isolates (labeled A1 and A2) were used as comparison strains. The vancomycin-resistant isolates were obtained from the Laboratory of Clinical Bacteriology at Karolinska Hospital in Huddinge, Stockholm, Sweden. Additionally, we investigated the antibiotic susceptibility of 34 endodontic isolates obtained from infected dental root canals. These endodontic isolates were acquired between the years 2000 and 2014 by the Laboratory of Clinical Oral Microbiology of the Department of Odontology, Umeå University, Sweden. These isolates were collected, de-identified, and detached from any clinical metadata with the aim of constituting the clinical isolate library of the Department. The absence of metadata and anonymization of the collection process prevent the isolates from being traced back to individual patients. These isolates are employed for educational and research purposes only. 

Isolates were selected following previously described procedures [5]. Briefly, endodontic samples were collected from post-treatment infected dental root canals, where an endodontic retreatment was indicated. Endodontic samples were plated onto bile/esculin/sodium azide agar plates specifically formulated for the isolation of group D streptococci. Esculetin-positive colonies (black colonies ensuing from esculin hydrolysis) underwent gram staining and microscopic examination. From these, only Gram-positive diplococci were further subjected to biochemical assays. Specifically, *E. faecalis* cells were selected based on their pyroglutamyl aminopeptidase (PYR) and leucine aminopeptidase (LAP) activities using methy-lumbelliferyl-associated substrates. PYR/LAP-positive isolates, which were taxonomically classified as *E. faecalis,* were further validated using species-specific quantitative PCR with the primers F: 5′- CCGAGTGCTTGCACTCAATTGG-3′ and R: 5′-CTCTTATGCCATGCGGCATAAAC-3′, which amplify a 138 bp amplicon on the 16S rRNA gene [74]. The isolates were stored in 20% skimmed milk at −80 °C.

For the current investigations, all *E. faecalis* strains and isolates were routinely cultured onto Müller–Hinton fastidious (MH-F) agar plates containing 5% defibrinated horse blood and 20 mg/L β-nicotinamide adenine dinucleotide (β-NAD) (Karolinska University Laboratory, Huddinge, Stockholm, Sweden) at 35 °C. MH-F agars supplemented with vancomycin (6 µg/mL) were used to maintain the two vancomycin-resistant isolates A1 and A2.

### 4.2. In Vitro Antimicrobial Susceptibility Testing

*E. faecalis* cells were inoculated from frozen stocks onto MH-F agar plates and incubated overnight at 35 °C. Inocula were prepared by picking colonies from overnight agar cultures and suspending them in 1 mL of phosphate-buffered saline (PBS). For standardization and culture quality purposes, only agars that were 2 to 4 passages old were used to generate the inocula to be tested. Several morphologically similar colonies were collected to avoid atypical variants. The bacterial density of the suspension was spectrophotometrically adjusted to OD_600 nm_ 0.2 (equivalent 0.5 McFarland, approx. 1.5 × 10^8^ CFU/mL) (UV-1800 spectrophotometer, Shimadzu, Kyoto, Japan) and homogenously plated onto MH-F agars using sterile cotton swabs.

The minimum inhibitory concentration (MIC) of several antibiotics was screened using Etest gradient strips (bioMérieux Sweden AB, Askim, Sweden). Antibiotic molecules included amoxicillin, clindamycin, vancomycin, tigecycline, linezolid, and daptomycin. The Etest strips were applied immediately after inoculation on MH-F agars, and the plates were incubated at 35 °C for 20 h (with a 24 h incubation specifically for vancomycin strips to detect resistance in inducible *vanA* carrier strains). Results were interpreted using the publicly available breakpoint tables on EUCAST website (www.eucast.org) (URL accessed on 30 November 2023). Specifically, *E. faecalis* isolates were categorized as “S—Susceptible” when their MIC was within the range of the EUCAST’s ECOFF. Isolates were categorized as “I—Susceptible, Increased exposure” when their MIC was equal to the EUCAST breakpoint, which would translate into a high likelihood of therapeutic success if the concentration of the antibiotic was adjusted. Finally, isolates were categorized as “R—Resistant” when their MIC was higher than the EUCAST breakpoint.

## Data Availability

The data presented in this study can be made available from the corresponding authors upon reasonable request.

## Supplementary Materials

The following supporting information can be downloaded at https://www.mdpi.com/article/10.3390/antibiotics13010018/s1, Table S1: MIC values of six antibiotics against various *E. faecalis* isolates.
