# Supplementary material for: Profiling Antibiotic Susceptibility among Distinct Enterococcus faecalis Isolates from Dental Root Canals
Source: Antibiotics (Basel). 2023 Dec 24;13(1):18. doi: 10.3390/antibiotics13010018 (PMC10812444; doi:10.3390/antibiotics13010018)
Supplement: Supplementary file 1 [file antibiotics-13-00018-s001.zip › antibiotics-2775823-supplementary.pdf]

Supplementary Table S1: MIC values of six antibiotics (amoxicillin, clindamycin, vancomycin, tigecycline, linezolid, and daptomycin) against various *E. faecalis* isolates.

	Amoxicillin (µg/ml)	Vancomycin (µg/ml)	Clindamycin (µg/ml)	Tigecycline (µg/ml)	Linezolid (µg/ml)	Daptomycin (µg/ml)
MIC breakpoints	≤4	≤4	N/A	≤0.25	≤4	≤4
**ATCC 29212**	1	3	6	0.19	2	2
**A1**	1	≥256	≥256	0.38	2	1
**A2**	0.75	≥256	≥256	0.38	2	0.75
**UmID1**	1.5	1.5	8	0.19	3	1
**UmID2**	2	1.5	≥256	0.25	1.5	1.5
**UmID3**	1.5	1.5	4	0.19	2	0.75
**UmID4**	1	2	12	0.25	2	1.5
**UmID5**	0.75	3	24	0.25	2	2
**UmID7**	0.75	2	4	0.19	3	3
**UmID10**	1	3	24	0.25	2	3
**UmID11**	1	2	≥256	0.125	1.5	0.75
**UmID12**	0.5	2	3	/	/	/
**UmID13**	1	3	32	0.19	3	2
**UmID15**	0.75	2	8	0.125	3	0.75
**UmID17**	0.75	3	32	0.19	3	3
**UmID18**	0.75	2	3	/	/	/
**UmID23**	1	4	24	0.19	3	3
**UmID24**	0.75	1	4	/	/	/
**UmID27**	1	3	≥256	0.25	2	1
**UmID30**	0.5	1.5	24	0.25	2	1
**UmID31**	1	3	16	0.25	3	1.5
**UmID33**	1	2	≥256	0.19	1.5	1
**UmID34**	0.75	1	3	/	/	/
**UmID35**	1	1	4	/	/	/
**UmID37**	0.75	1.5	2	/	/	/
**UmID40**	1	1	24	0.094	2	0.75
**UmID42**	1.5	2	8	0.125	2	1
**UmID43**	1.5	3	12	0.125	3	1.5
**UmID44**	1.5	1	16	0.094	2	1.5
**UmID46**	1.5	3	32	0.125	3	3
**UmID47**	1	3	32	0.125	3	1
**UmID49**	1.5	3	12	0.094	3	2
**UmID51**	1.5	3	12	0.19	4	1
**UmID53**	1.5	3	8	0.125	3	2
**UmID54**	1.5	3	24	0.125	2	0.75
**UmID55**	1	1	1.5	/	/	/
**UmID56**	1	4	24	0.064	3	1

Light red: denotes resistant MICs above the breakpoint. **Orange:** denotes isolates exhibiting clindamycin MICs above that of the ATCC 29212 reference strain. **Grey:** denotes MICs that match the breakpoint for the respective antibiotic, and that could be considered "I – Susceptible, increased exposure" by the EUCAST.
